# Challenges and Strategies in Nursing Leadership: A Qualitative Study on Leaders in Mental Health Care

**DOI:** 10.3390/nursrep14040288

**Published:** 2024-12-10

**Authors:** Else Marie Lysfjord, Edith Roth Gjevjon, Siv Skarstein

**Affiliations:** 1 Faculty of Nursing and Health Sciences, Nord University, 7804 Namsos, Norway; 2 Department of Bachelor Education, Lovisenberg University College, 0456 Oslo, Norway; edith.gjevjon@ldh.no; 3 Department of Bachelor Education, UiT The Artic University of Norway, 9404 Harstad, Norway; 4 Department of Nursing and Health Promotion, Faculty of Health Sciences, Oslo Metropolitan University, 0130 Oslo, Norway; siv.skarstein@oslomet.no

**Keywords:** leadership challenges, leadership development, mental healthcare, mentoring programs, new and experienced leaders, nursing leadership, strategic leadership, support networks

## Abstract

Aim: To explore the challenges and strategies among new and experienced nursing leaders in mental healthcare; furthermore, to identify factors that support or hinder their leadership roles. Background: Strong nursing leadership is crucial for the quality of patient care and is associated with higher job security and better patient outcomes. Understanding what factors contribute to effective leadership is essential for the development of future leaders. Methods: A qualitative study was conducted through interviews with 20 nursing leaders in mental healthcare in Norway, including 10 new leaders (<2 years in the role) and 10 experienced leaders (>10 years in the role). Data were analyzed using a six-step thematic analysis. Results: New leaders set high standards and faced demanding tasks, which made the role stressful. They experienced uncertainty and self-doubt about their effectiveness and expressed a need for support from mentors or colleagues. Experienced leaders focused on strategic leadership, task prioritization, and employee motivation, emphasizing the importance of being inspirational, patient, and accessible. Discussion: This study highlights the different challenges faced by new and experienced leaders in mental healthcare. New leaders need support to build confidence and manage the demands of their roles, while experienced leaders benefit from their strategic approach and ability to motivate staff. Conclusions and implications for nursing and/or health policy: The findings suggest that mentoring programs and support networks are essential for developing and motivating nursing leaders. New leaders should receive support to overcome self-doubt and stress associated with their roles. Experienced leaders can, through being mentors, expand their strategic skills and increase own insight and abilities regarding leadership. These insights have significant implications for health policy, which should include resources and programs aimed at supporting leadership development in nursing.

## 1. Introduction

Leadership is one of four pillars of the nursing profession besides clinical practice, education, and research [1]. Nursing leadership involves organizing, directing, and managing nursing services with the goal of ensuring efficient, safe, and high-quality patient care. The leadership is responsible for supporting and developing nurses, as well as implementing strategies that promote positive patient outcomes [2]. Higher expectations for healthcare services increases the need for nursing leadership within healthcare organizations [3]. Indeed, strong leadership qualities in nursing leaders are associated with greater job satisfaction, reduced turnover, positive intentions among nursing staff, and also improved patient outcomes [4,5]. Furthermore, leadership styles highly influence registered nurses’ (RN) professional development, work environment, professional practice, motivation, and job behavior [6,7]. Gaining insight into factors that contribute to excellence in nursing leadership is fundamental to ensure a future supply of nursing leaders, who can positively influence outcomes for both healthcare providers and patients [8,9]. Leadership that cultivates a collaborative and supportive working environment is associated with higher staff satisfaction [5,10]. Leadership that focusses on participation, empowerment, reward, community, fairness, workload, and values, all of which can be guided by management, has been found to reduce turnover among mental health nurses and also increase patients’ satisfaction with the treatment they receive [11,12,13]. Supporting and developing RNs as leaders to manage such leadership qualities will be important to ensure the good quality of the nursing service [9,10,14].

Strategic leadership is significant for recruiting, motivating, and retaining RNs and nurse leaders to ensure high-quality healthcare for our society [15,16]. RN burnout is reported globally, and nurse leadership plays a significant role in reducing burnout. Competent leaders can reduce RN burnout through empowering and promoting RN engagement, applying authentic and transformational leadership, and creating a healthy work environment.

The major influencing leadership styles include authentic and transformational leadership [17]. It is also the case that the more satisfied RNs are with their supervisors and management, the less they experience burnout [18]. Nevertheless, when RNs are less satisfied with leadership, interpersonal strain increases, and counterproductive work behaviors increases, reducing patient satisfaction with the care received. Furthermore, if RNs have a poor perception of leadership, their cynicism increases, and the quality of care perceived by patients decreases [19].

Studies imply that psychiatric nurses, also named mental health nurses (MHN), who are leaders in mental healthcare, feel they lack a mandate to lead their units. This can lead to uncertainty in their leadership role [20,21]. Sundberg (2022) suggests that this might be a result of a feeling of responsibility and meaningfulness, mixed with a sense of powerlessness and uncertainty. Such ambivalence in the leadership role is prominent among nurses in leading positions [20]. An earlier study shows that nurses who are new in the leadership position struggle with professional confidence as a leader and that they are unsure of the expectations and requirements of their new role [1].

RNs are often recruited to leadership roles following extensive clinical practice within their own organization, but this has been shown to result in a challenging role transition [21]. Therefore, the aim of this study is to describe leadership roles from the perspectives of both new and experienced RNs in leading positions within mental healthcare to capture different stages in this process. Such knowledge might facilitate future support for RNs as leaders.

For this study, rooted in the context of the Norwegian healthcare system, spanning both community and specialist levels, the following research questions were formulated:

1. How do Registered Nurses (RNs) new to leadership positions and RNs with extensive leadership experience, respectively, perceive their roles as leaders?
2. What are the possible differences in the primary areas of attention in the leadership role for RNs new to leadership positions, as well as RNs who are experienced leaders?

To explore the research questions of this study, we conducted qualitative structured interviews with both new and experienced leaders in the field of mental health. The selection, data collection, and analysis of data are described in the methods section. The findings are presented in a separate section titled Findings. In the Discussion section, the study’s findings are examined in relation to relevant research. Furthermore, ethical considerations and the study’s strengths and limitations are addressed, before concluding with the implications for practice.

## 2. Methods

To gain a deeper insight into RN leaders’ experiences, reflections, and perspectives regarding their leadership role, we conducted a qualitative descriptive study. A qualitative descriptive approach allows for the data gathered to stay closely connected to the phenomenon under investigation [22,23]. In this case, the roles of new and experienced RN leaders within mental health settings. All of the authors are experienced researchers within the field of mental health or nursing. The researchers have also contributed to the scholarly literature on mentoring. This literature has been utilized in structuring and curating the academic content of the mentor program. An interview guide for qualitative research was developed based on the literature review. Furthermore, participants in the mentor program were informed about this follow-up research and obtained consent for participation and publication. A list that links personally identifiable information to a code list is maintained at one researcher’s university.

## 3. Sample and Context

The study sample consisted of RNs participating in a mentoring program provided by the Norwegian Nurses Organization (NNO). As part of the pilot testing of the mentorship program, a follow-up study addressing several aspects of leadership, in addition to exploring the effects of the “Mentorship Program” pilot, was conducted. The interviews for this study were conducted at the start and the end of the program. All participants were invited and accepted to participate in the study. Twenty nurse leaders in the field of mental health from different parts of Norway were included in this study. In this study, “leader” refers to nurses who hold a formal leadership role. Ten had been in their current leadership position for less than two years, hereafter referred to as “new” leaders. The other group consisted of ten leaders who had been in leadership positions for more than ten years, hereafter referred to as “experienced” leaders. Experienced nurse leaders served as mentors, whilst newly appointed nurse leaders were mentees. All participants were registered nurses (RNs) and had a background from clinical work within mental health. Fifteen of the participants were female and five were male.

## 4. Data Collection

The data collection took place during the autumn of 2021. In this study, we chose to conduct individual interviews, which allow for a deeper exploration of personal thoughts, feelings, and experiences without the influence of other participants. This approach often results in more honest and open responses [24]. The research team, consisting of one professor, two associate professors, and three nursing managers, all with nursing backgrounds, developed a semi-structured interview guide. They conducted a thorough review of the literature focusing on nurses in leadership roles, which formed the basis for crafting relevant questions. The aim was to explore the experiences of both new and experienced nurse leaders and to identify factors perceived as either facilitators or barriers to their success. The guide encouraged nurse leaders to reflect on their thoughts, feelings, and skills related to their leadership roles. The same interview guide was used for both new and more experienced leaders. In the interview guide, interviews began with background questions and concluded with questions about the nurses’ reflections regarding being in a leadership position. Each theme began with a question such as “What do you think…”, for example, “What do you think is most challenging for new leaders in the field of mental health and substance abuse?” and “What are your future ambitions as a leader?” Further questions were based on the informants’ responses. Concrete examples of situations, clarifications, and further elaborations were requested.

Telephone interviews were conducted individually by a hired professional interviewer, each lasting approximately 45 min. The interviewer possessed expertise in research and interviewing techniques and had no prior relationship with the participants. Notes and quotes were systematically recorded during and after each interview, allowing for the participants to clarify and expand upon their statements. This approach aimed to identify and rectify any misunderstandings or ambiguities. The interviews were conducted in the participants’ native language, Norwegian. They were transcribed in Norwegian and then translated into English by the research team. The members of the research team had a good command of the English language.

## 5. Data Analysis

The data material was divided into two groups: new leaders (ten interviews) and experienced leaders (ten interviews). The interviews from each group were analyzed separately. Later, the identified meaningful units from the respective two groups were then contrasted. Previous research suggests that ten interviews may be sufficient to provide a comprehensive understanding of a topic. A key source for this is the study by Guest, Bunce, and Johnson [25], which found that data saturation mostly is reached from within the first six to twelve interviews. Their research demonstrates that once saturation is achieved, little to no new information is added through additional interviews, supporting the notion that a sample size of around ten can be adequate to gain the necessary insights into the subject matter [25]. Additionally, the decision to conduct ten interviews per group was based on the existing literature, which supports this number as sufficient to achieve saturation in similar studies [26]. All participants were interviewed, although saturation in the material was achieved after six interviews with adepts and after seven interviews with mentors.

To analyze the interview data, we employed thematic analysis in six steps as outlined by Braun and Clark [27]. In the first step of the analysis, the authors read each interview separately. In the second step, each of these two authors coded the data and collected relevant content and quotes under each code. The codes, content, and quotes/statements were then organized in a summary table. In the third step, the authors compiled statements into potential themes. Subsequently, they reviewed the themes to ensure their logical alignment with the entire interview text material. The authors then defined and named each theme. After the first three steps of the analysis process, the authors met to discuss and coordinate their analyses and results. In this fourth phase, the authors reached agreement on the main themes. Beforehand, the authors had decided that, in cases of disagreement or ambiguity, the authors would revisit the original material and discuss the understanding and interpretation until a consensus was reached. In the fifth step of the analysis, the main themes were named. Furthermore, meaningful quotes to underpin the themes were discussed and selected. The process concluded with the production of an analysis report.

To maintain the anonymity of the participants, statements from the new leaders are labeled with the letter “A” followed by a number indicating the interview number. For the experienced leaders, statements are marked with the letter “M” and a corresponding interview number. This labeling system ensures that the responses are identifiable by category (new or experienced leaders) without revealing the identity of the individual informants.

Table 1 shows an example from the thematic analysis: the key statements from the new and experienced leaders, the identified meaningful units, the assigned codes, and the corresponding themes from the analysis of interviews with the new leaders.

## 6. Ethical Considerations

The participants received both written and verbal details regarding the research and gave written consent to be interviewed. They were informed that it was optional to participate and that they could withdraw from the study at any time with no risk of repercussions.

Neither the authors nor the interviewer possessed any affiliations with the participants that might influence their involvement. To safeguard participant confidentiality, pseudonyms or numerical identifiers were employed when citing quotations in this paper.

The design of the current study adhered to the general guidelines for research ethics set forth by the Norwegian National Research Ethics Committee. Additionally, the research protocol was assessed by the SIKT—Norwegian Agency for Shared Services in Education and Research (project number 386161).

## 7. Trustworthiness

Trustworthiness in qualitative research is assessed through four key criteria: credibility, dependability, confirmability, and transferability [28]. All eligible participants were interviewed, which strengthens the credibility of the results. In addition, the use of a structured interview guide and an independent and consistent interviewer with no pre-understanding of the topic under study further enhanced credibility. Dependability was addressed by collecting data soon after the program was ended, ensuring that participants’ experiences were fresh and consistent. Confirmability was enhanced, as all authors engaged in the data analysis process both independently and collaboratively. In this study, transferability was supported by a detailed description of the context, and a thorough, clear explanation of the data collection and analysis methods. The Standards for Reporting Qualitative Research (SRQR) [29] were employed to maintain transparency and quality throughout the research and reporting phases.

## 8. Findings

Participation in the mentorship program demonstrated that the participants in this study were inherently engaged leaders. All possessed leadership competencies, although the novice leaders had short tenure while the experienced leaders had extensive tenure.

From the analysis of the interviews with the ten new leaders, three main themes were identified: new leaders set high standards for themselves and found the leadership position stressful; new leaders felt vague and were doubtful regarding their own ability; and new leaders lacked sufficient professional support.

**Theme** **1.** *New leaders set high standards for themselves and find the leadership role demanding and stressful*.

One of the main findings of this study was that nurses in leadership positions within the field of mental health and substance abuse set high standards for themselves in leadership positions. One of the new leaders said, “*I want to be a role model for the employees*” (A14).

Furthermore, the leader’s responsibility to appear distinct, democratic, facilitate cooperation, strengthen employees, and point out the direction for further work is emphasized. The following statements from the new leaders underscored their experience of leadership responsibility:

“*A leader must facilitate interdisciplinary cooperation*” (A9).

“*A leader must be clear, democratic, and open*” (A9).

The new leaders emphasized the care of employees, primarily highlighting that central leadership qualities include being attentive, available, supportive, and helping the employees. Statements by the new leaders describing these qualities included the following:

“*A leader must be supportive and attentive*” (A11).

The findings show that the informants feel unprepared for the leadership task, while several reported felt overwhelmed by workload. Examples from the interviews illustrating the informants’ experiences included the following:

“*Employee follow-up, delegation, task prioritization, handling sick leave, and personnel recruitment are demanding tasks. This requires time and resources, as well as training and support, which are lacking*” (A7), and “…*Often thrown into the job, expected to know everything immediately*” (A13).

In addition to feeling overwhelmed by workload, the informants also experienced other aspects of leadership responsibility as challenging, such as conflict management. One informant expresses it as follows:

“*I find conflicts difficult and challenging*” (A10).

**Theme** **2.** *New leaders feel vague and doubt their abilities*.

Several of the new leaders expressed a desire to gain increased control over both themselves and the situations they encountered in their new role. This was expressed, for example, as follows:

“*As a leader, I need control*” (A9).

New leaders reported experiencing doubt regarding their ability to handle their leadership role effectively and a desire to be independent as leaders, like this quote illustrate:

“*I want to be self-reliant as a leader*” (A14).

But at the same time, they felt the need to develop greater calmness and confidence in their role, expressed as follows:

“*I need to feel calmness and confidence in my leadership role*” (A14). 

This doubt seemed to lead to a desire to achieve a sense of security and stability in their leadership role, as expressed by another of the new leaders: 

“*I want to become a more distinct leader*” (A10).

To sum up this theme, the new leaders expressed that they felt uncertain due to the increased burden of responsibility and the new challenges they faced. A feeling of being overwhelmed by the expectations of others and the complex nature of the leadership role was articulated.

**Theme** **3.** *New leaders lack sufficient professional leadership support*.

Most of the new leaders faced challenging decisions and responsibilities that felt overwhelming. A feeling of loneliness arose due to a lack of management and leadership support to share experiences and concerns with, as well as a lack of support from colleagues or subordinates. In today’s complex and dynamic work environment, the need for continuous learning and networking is crucial to developing effective leadership. Leaders often seek forums where they can share knowledge and experiences to enhance their skills and approaches. One of the new leaders said,

“*I need a network where I can discuss and gather knowledge from others*” (A15).

Another formulated the following: 

“*I need to exchange leadership experiences with others to find my own path*” (A11), “*I need to exchange leadership experiences with others to find my own way of leading*” (A11), and “In my leadership role, I receive little guidance as a leader” (A14).

From the analysis of the interviews with the ten experienced leaders, it was clear that these leaders were highly focused upon the tasks that they considered to be crucial for nurses in leadership positions. Within the two main themes which were identified, four subthemes were integrated. The two main themes, presented as Theme 4 and Theme 5 are as follows: for experienced leaders, strategic leadership is a vital task, and sustaining and motivating employees is imperative for success.

**Theme** **4.** *Strategic leadership is vital for experienced nurse leaders*.

Experienced leaders emphasized the importance of strategic leadership in the nursing profession. One informant said,

“*The most important task it’s to lead the organization towards its goals*” (M10).

Experienced leaders stressed the importance of motivating and guiding individuals to perform their best both individually and as part of a team, focusing on the organization’s goals and direction. Informant number 18 expressed it in the following manner:

“*A leader must motivate employees to perform their best individually and as a team towards the organization’s goals and direction*” (M18).

A significant point raised was about prioritization. During diverse tasks and responsibilities, a leader must be able to identify and focus on what is most crucial. As one of the experienced leaders put it: 

“*A leader must have the ability to prioritize*” (M18). 

This ability to discern and decide what takes precedence can be the difference between success and failure. Similarly, persistence was highlighted as a vital quality in leaders. Leaders often need to steer their teams towards long-term strategic goals, which can be fraught with challenges and obstacles. Despite these, leaders must stay the course and remain steadfast. This sentiment is encapsulated in the words of one informant, who stated,

“*A leader must be persistent to achieve strategic goals*” (M17).

This persistence can fuel the consistent efforts needed to realize strategic objectives, further underscoring its importance in effective leadership.

Experienced leaders emphasized the importance of a leader being willing to make unpopular decisions, daring to face adversity, as evidenced by this statement from the informant: 

“*A leader must be willing to make unpopular decisions*” (M17).

The experienced leaders saw strategic leadership as significant in the nursing profession, focusing on guiding both individuals and the organization towards common goals, whilst handling challenges and making tough decisions with strength and determination.

**Theme** **5.** *A resilient leader motivating employees is an imperative for success*.

Experienced leaders highlighted several key qualities crucial for effective leadership in healthcare. Firstly, it is essential for a leader to be resilient, understanding the ability to handle challenges with strength and endurance. Experienced leaders described these qualities as follows:

“*A leader must dare to face adversity*” (M18) and “*A leader must be resilient*” (M12). 

Furthermore, the importance of being inspiring and able to align people towards a common goal through clear vision and motivation was emphasized. Informant M10 conveyed the following:

“*A leader must inspire, getting people to move in the same direction*” (M10).

Experienced leaders further emphasized that being a caring, secure, and supportive leader is crucial for creating a positive work environment, where employees feel valued and supported. One of the informants described it as follows:

“*A leader must care about their employees, achieving good interaction to make going to work enjoyable*” (M12).

Lastly, it is important for a leader to be accessible to their employees. One of the informants said,

“*A leader must be present to build trust and engagement*” (M17).

These qualities were seen by the informants as fundamental in creating an efficient and pleasant work culture and leading the team towards common goals.

## 9. Discussion

The aim of this study was to gain a deeper insight into the leadership role of new and experienced RN leaders within mental healthcare. This study highlights the leadership role from two different perspectives: those of new and experienced leaders. Few studies have addressed this issue, and few have specifically examined the leadership role within the field of mental health. Our findings show that new leaders set high standards for themselves, find the leadership position demanding and stressful, feel vague as leaders, and miss leadership support and being part of a leadership network. In addition, they are apprehensive regarding their own ability to manage their leadership role. The experienced leaders were more focused on strategic leadership as a vital task for nurse leaders, as well as the importance of sustaining and motivating employees. These issues were barely mentioned in the interviews with the new leaders.

### 9.1. Focus on Own Performance Versus Strategic Focus

As a nurse in a leadership position, balancing attention and a focus on personal performance and strategic focus is crucial [30]. On one hand, personal performance involves continuously improving clinical, leadership, and personal skills such as communication abilities and problem-solving capacities [30,31,32]. On the other hand, a strategic focus is equally important [32]. This involves understanding the larger organizational goals, planning for future challenges, and aligning team efforts with the broader vision of the healthcare organization [33,34]. While a focus on personal performance ensures excellence in immediate nursing tasks and team management, a strategic focus positions the nurse leader as an influential driver of long-term organizational success. Both aspects are integral to the role of a nurse in a leadership position, creating a balanced approach to effective healthcare leadership.

### 9.2. Transition and Development

New leaders grapple with a sense of ambiguity regarding their leadership role, and some seem to doubt their ability to effectively manage their leadership responsibilities [21]. Higher levels of self-efficacy have been found to mediate the effects of work stress on job burnout among nurses in leadership positions [35]. Reduced self-efficacy may result in a prolonged period of adjustment and learning. In the adjustment and learning period, the new leader has a high focus on understanding their own role, enhancing personal leadership skills, and building confidence [36,37]. This might cause uncertainty within the staff, which can lead to a loss of focus on work tasks and patient care. This highlights the importance of support systems and training for new leaders, ensuring a smooth transition that maintains the quality of service [9,37]. On the other hand, experienced leaders, having already navigated these initial challenges, tend to focus more on maintenance and motivation of their teams [38]. They understand that the key to success lies in cultivating a motivated, engaged, and satisfied team and their focus seems to have shifted from their own personal performance to team performance [39]. These differing focuses will yield differing results. For new leaders, emphasis on personal growth and learning can lead to rapid skill development and a steep learning curve. For experienced leaders, their focus on team motivation can result in increased team productivity and improved work morale. Nevertheless, it is also important for experienced leaders to make sure they always have an opportunity to develop as leaders [40]. The growth and learning that occurs for new leaders sets the foundation for the team-focused approach that characterizes successful, experienced leaders, and both stages are vital parts in the leadership journey. This journey from self-focused to team-focused is a key aspect of leadership development in any field, including nursing [41]. Nevertheless, being supported by a qualified management or/and a mentor might underpin an effective transition from a new to a experienced leader [9,21].

### 9.3. Experienced Leaders’ Roles in Supporting New Leaders: A Pathway to Organizational Success

Stepping into a leadership position as a new leader can, as the new leaders express, often be demanding and stressful [21,42]. The absence of sufficient leadership support and a robust leadership network can exacerbate these challenges [21]. Therefore, it is encouraging to see that experienced leaders understand the importance of strategic leadership, including taking care of employees like new nurse leaders. Experienced leaders focus on sustaining and motivating new nurse leaders, which implies that they are committed to supporting their new colleagues in their journey towards effective leadership. This approach is positive, as it not only aids the personal growth of new leaders but also stimulates organizational success [19,37]. By identifying and addressing the needs of nurses who are new in the leadership position, experienced leaders play a crucial role in fostering a supportive environment [19,30]. This helps new leaders transition smoothly into their roles, and this will again ultimately contribute to the overall success of their common healthcare organization [37].

## 10. Strengths and Limitations

The homogeneity of the participants in terms of their educational backgrounds was a noted limitation. The inclusion of more male nurse leaders could have expanded our understanding of the topic, possibly unveiling gender-related differences in leadership experiences. Gender differences could offer interesting insights, but this was not an aim of this study. However, our sample consisted of all participants in the mentor program, and there was no male–female allocation by the NNO. The participants’ awareness of the study’s focus on the utility of mentorship may have influenced their responses. Due to the authors not having English as their first language, it cannot be overlooked that the translation into English may have led to ambiguities. However, a professional editor has supported us with the language. The participants in this study are all engaged in leadership development, as evidenced by their interest in participating in the mentoring program. Consequently, the results may not necessarily be universally applicable or transferable to all leaders within the field of mental health. Despite these limitations, the sample’s strength lies in geographic diversity and representation of different levels within the health service. This study includes both new and experienced leaders, providing a broader insight into how the leadership role evolves over time, as well as the unique challenges and coping strategies for each group. The insights into the transition challenges faced by new leaders highlight critical support needs during the transition from nurse to leader. This knowledge holds practical value for the development of leadership training programs and recruitment strategies within nursing leadership.

## 11. Conclusions and Implications for Nursing and/or Health Policy

The research provides valuable insights into how Registered Nurses (RNs) new to leadership positions and RNs with extensive leadership experience within mental healthcare perceive their roles as leaders. Furthermore, some important targets for supporting and motivating new nurse leaders are identified.

New leaders often grapple with self-doubt, high self-imposed standards, and a sense of ambiguity about their roles. They express a need for greater leadership support and networking opportunities. Conversely, experienced leaders emphasize strategic leadership and employee motivation as essential factors for success. These differences may reflect the natural progression of leadership development, with new leaders concentrating on personal growth and skill acquisition, and experienced leaders shifting their attention towards team growth and strategic planning.

This study reveals that new nurse leaders in mental health and substance abuse face challenges such as stress, uncertainty, and a lack of professional support. This underscores the need for improved support programs, including mentorship and networking opportunities, to help new leaders build confidence and manage demanding situations. For experienced leaders, strategic leadership and the ability to motivate staff are crucial for success.

The findings suggest that leadership development programs should focus on assisting new leaders with the transition into their roles, while experienced leaders can benefit from further development of strategic and motivational skills. Strengthening leadership capabilities in this way can enhance both the leaders and the organizations they manage, contributing to a healthier work environment and improved patient care.

Allocating resources for leadership development is essential to ensuring effective nursing leadership, which is critical for both staff well-being and patient outcomes.

Our dataset is available on request from the authors. The raw data supporting the conclusions of this article will be made available by the authors on request.

## Figures and Tables

**Table 1:** The table below presents the key statements from the new and experienced leaders, the identified meaningful units, the assigned codes, and the corresponding themes from the analysis of the interviews. Themes 1, 2, and 3 represent the new leaders; themes 4 and 5 represent the experienced leaders.

Statement	Meaning	Code	Theme
I want to be a role model for the employees (A14)	Aspiring to be a role model	Role model	
A leader must facilitate interdisciplinary cooperation (A9)	Emphasizing cross-disciplinary collaboration	Facilitate	Theme 1: New leaders set high standards for themselves and find the leadership role demanding and stressful.
A leader must be supportive and attentive (A11)	Importance of being supportive and attentive	Supportive	
A leader must be clear, democratic, and open (A9)	Prioritizing clarity, democracy, and openness	Clear	
I find conflicts difficult and challenging (A10)	Perceiving conflicts as demanding	Conflicts	
Employee follow-up, delegation, task prioritization, handling sick leave, and personnel recruitment are demanding tasks. This requires time and resources, as well as training and support, which are lacking (A7)	Overwhelmed by administrative duties	Overwhelmed	
…Often thrown into the job, expected to know everything immediately (A13)	Feeling unprepared for leadership role	Unprepared	
I want to become a more distinct leader (A10)	Aspiring to enhance leadership distinctiveness	Distinct	Theme 2: New leaders feel vague and doubt their abilities.
I want to be self-reliant as a leader (A14)	Striving for self-reliance	Self-reliant	
As a leader, I need control (A9)	Expressing a need for control	Control	
I need to feel calmness and confidence in my leadership role (A14)	Need for calmness and confidence	Calmness and confidence	
I need a network where I can discuss and gather knowledge from others (A15)	Need for a professional network	Professional network	Theme 3: New leaders lack sufficient professional leadership support.
I need to exchange leadership experiences with others to find my own path (A11)	Exchange leadership experiences	Exchange experiences	
In my leadership role, I receive little guidance (A14)	Lack of leadership guidance	Guidance	
The most important task it’s to lead the organization towards its goals (M10)	Emphasizing strategic leadership	Strategic	Theme 4: Strategic leadership is vital for experienced nurse leaders.
A leader must be persistent to achieve strategic goal (M17)	Persistent	Strategic	
A leader must motivate employees to perform their best individually and as a team to-wards the organization’s goals and direction (M18)	Fostering peak performance	Performance	
A leader must have the ability to prioritize (M18)	Emphasizing prioritization	Prioritize	
A leader must be willing to make unpopular decisions (M17)	Acknowledging need for unpopular decisions	Unpopular	
A leader must dare to face adversity (M18)	Importance of facing adversity	Adversity	Theme 5: A resilient leader motivating employees are imperatives for success
A leader must be resilient (M12)	Emphasizing resilience	Resilient	
A leader must inspire, guiding everyone in the same direction (M10)	Encouraging motivation and inspiration	Inspire	
A leader must care about employees for positive interaction and work enjoyment (M12)	Emphasizing engagement and interaction	Caring	
A leader must be present to build trust and engagement (M17)	Emphasizing leadership presence	Presence	

## Data Availability

The raw data supporting the conclusions of this article will be made available by the authors on request.

## Public Involvement Statement

No public involvement in any aspect of this research.

## Guidelines and Standards Statement

This manuscript was drafted against the Standards for Reporting Qualitative Research (SRQR) for Qualitative Research.
